# Molecular Mechanisms of Parturition

**DOI:** 10.1155/S1064744997000161

**Published:** 1997

**Authors:** F. Ferré

**Affiliations:** U. 361 INSERM Université René Descartes Pavilion Baudelocque, 123, Boulevard de Port-Royal Paris 75014 France

## Abstract

The initial signal for triggering human parturition might be fetal but of trophoblastic origin. Concomitantly, this placental signal would have as its target not only the uterus but also the fetus by activating its hypothalamo-pituitary-adrenocortical axis. The latter would represent a second fetal signal which, at the fetomaternal interface, would amplify and define in time the mechanisms responsible for the onset of labor, implying changes in the myometrial and cervical extracellular matrix associated with the accession of the contractile phenotype for myometrial cells. At each phase of these processes in the utero-feto-placental system, the nature of these signals remains to be identified. Is there a single substance, or rather, and more likely, a combination of several?

We appear to be in the presence of dynamic systems of a neuro-immuno-hormonal type which are difficult to describe. Nevertheless, steroid hormones appear to coordinate their successive equilibriums until they become irreversible. Such irreversibility constitutes the essential sign of parturition.


Infectious Diseases in Obstetrics and Gynecology 5:98–105 (1997)
© 1997 Wiley-Liss, Inc.

### Molecular Mechanisms of Parturition

F. Ferré

*U. 361 INSERM, Université René Descartes, Hôpital Cochin, Paris, France*

ABSTRACT

**The initial signal for triggering human parturition might be fetal but of trophoblastic origin. Concomitantly, this placental signal would have as its target not only the uterus but also the fetus by activating its hypothalamo-pituitary-adrenocortical axis. The latter would represent a second fetal signal which, at the fetomaternal interface, would amplify and define in time the mechanisms responsible for the onset of labor, implying changes in the myometrial and cervical extracellular matrix associated with the accession of the contractile phenotype for myometrial cells. At each phase of these processes in the utero-feto-placental system, the nature of these signals remains to be identified. Is there a single substance, or rather, and more likely, a combination of several?**

**We appear to be in the presence of dynamic systems of a neuro-immuno-hormonal type which are difficult to describe. Nevertheless, steroid hormones appear to coordinate their successive equilibriums until they become irreversible. Such irreversibility constitutes the essential sign of parturition. Infect. Dis. Obstet. Gynecol. 5:98–105, 1997.** © 1997 Wiley-Liss, Inc.

KEY WORDS
Human parturition, placenta, uterus, extracellular matrix, signaling factors, contractility

Although the act of "bringing a child into the world" is one of the essential keys to life and to the survival of the species, we cannot help but be amazed even today by the fact that most of the physiological mechanisms of human parturition remain to be elucidated. Giving birth is not a trivial act. Indeed, the pathologies associated with it, whose etiologies remain poorly understood, can expose both mother and child to varying types of grave complications. Premature birth, for instance, is an important unsolved health problem.

One of the reasons why the processes of human parturition remain to some extent enigmatic is that results of research utilizing animal models, which can then be transposed to humans, are, in fact, very rare. Indeed, there exists extreme species diversity, especially in endocrine balance, which conditions the maintenance of gestation. In women, one particularity would appear to lie in the absence of a correlation between the evolution of available parameters, such as the plasma level of steroid hormones or of many other effectors of uterine activity, and the onset of labor. In this respect, it is only recently that we have became aware of the specificity of the human species, as current data reveal a multitude of pertinent factors, be they genetic, hormonal or environmental. This complexity, also present in the mechanisms of action of these factors, has for the moment escaped any simplifying principle, whatever the level of analysis of the biological systems which are targets in the maternal-fetal system.

Nevertheless, although experimentation is limited by considerations of an ethical or practical nature, the availability of molecular biological techniques and of selective pharmacological tools, and the development of human cell models in the same way as those enabling better understanding of the different components of uterine contraction, should facilitate an approach to this intricacy.

The initial question raised is that of the hormonal determination of parturition. The second,

*Correspondence to: F. Ferré, U. 361 INSERM, Université René Descartes, Pavillon Baudelocque, 123, Boulevard de Port-Royal, 75014 Paris, France, E-Mail u361@cochin.inserm.fr

Received 1 October 1997

Accepted 21 October 1997

upon which we will place special emphasis, concerns the molecular mechanisms which, in the uterus, underlie the two phenomena which characterize parturition: the onset of regular, rhythmic myometrial contractions and dilatation of the cervix.

### HORMONAL DETERMINATION OF PARTURITION

Much data has been obtained concerning the mechanisms involved in the onset of parturition in several animal species.[1] Nonetheless, it must be admitted that some of these data have had the effect of momentarily preventing other approaches in humans. The most striking example is that of the ewe, with the discovery of an initial signal from the fetal brain leading to a drop in placental production of progesterone (which blocks uterine excitability) and an increase in production of estrogens (which promote uterine contractility).[2] For several years, a number of groups vainly sought the existence of identical mechanisms in the human species. At birth, the fetus, depending on the species, is confronted with extremely varied situations necessitating different competences. Its survival depends upon its degree of maturation in the broad sense of that term, since it can involve different functions. In the human species, where the fetus confronts extra-uterine life in a state of relative immaturity and dependence, a fetal signal is difficult to identify. In anencephalic fetuses, parturition occurs in a natural manner at term, but is slightly more dispersed in time than in normal fetuses. While a role for fetal membranes has been suggested, based on their capacity to locally control the metabolism of steroid hormones, prostaglandins and cytokines, studies are currently focusing on the placenta and in particular on its endocrine tissue, the trophoblast. One of the characteristics of this tissue is not only its extraordinary capacity for synthesis of a wide variety of biologically active substances such as steroid and polypeptide hormones, eicosanoids, neurotransmitters, vasoactive peptides, growth factors and cytokines, but also its capacity to express most of the receptors of these substances.[3] Each of these substances has varying degrees of pleiotropic functions. By controlling growth, differentiation, the synthesis of other signaling factors, immunity, and contractile activity at the level of numerous fetal and maternal targets, they participate in the development of the fetoplacental unit and in the adaptation by the maternal organism, primarily the uterus, at the gestational state.

The concept of fetoplacental unit, elaborated in the human species and up to now limited to biosynthetic, progesterone and estrogen pathways,[4] has recently been enriched by the discovery of other interactions. In the trophoblast, 11β-hydroxysteroid-dehydrogenase, which catalyzes the interconversion of cortisol into cortisone, would appear to play a role in triggering parturition. The decrease in the synthesis of placental cortisol would lead, at the end of pregnancy, to the activation of the fetal hypothalamic-pituitary-adrenocortical axis and synthesis of fetal cortisol. This would have the effect of accelerating pulmonary maturation of the fetus and of contributing, along with other hormones, to the production of placental corticotropin-releasing hormone (CRH), one of the effects of which is to activate uterine motility. This fetoplacental "dialogue," which begins early on in pregnancy, would reinforce the idea of the existence of a close relationship between fetal development and the duration of gestation in the human species and in certain primates.[5]

Of all the organs, it is the placenta which has the greatest species specificity in terms of its morphological, structural and functional characteristics.[6] Indeed, there exist several ways of optimizing the fetomaternal exchanges through the placenta. We might thus raise the question as to whether the species diversity noted in the mechanisms of the onset of parturition might not, in part, be linked to the mode of placentation (the human placenta is of a hemochorial type).

At the fetomaternal interface, structures other than the villous trophoblast are able to express a certain number of biologically active substances and their receptors: extravillous and invasive trophoblast, amniotic and decidual cells, and immune cells (macrophages, lymphocytes). The potentialities of expression of myometrial cells are also being examined at present. It is within the intervillous blood space, the circulation which is specific to the human fetomaternal interface, that some of these substances which control both the development of the fetus and uterine activity are found at high concentrations at the end of pregnancy.[7]

In this context, which favors regulations of a paracrine, autocrine and intracrine type, the role of each substance in the control of uterine activity is difficult to evaluate. This situation is accentuated at the site of placental insertion, where interactions between fetal and maternal cells are established which are both subtle and evolutive.[8] Indeed, it is in the human species that the invasion of uterine tissue by trophoblast cells is the most extensive, colonizing the wall of the spiral uterine arteries and reaching the inner layer of the myometrium.

Although we are uncertain whether it was chance alone which determined the diversity of the placental structure and that of the fetomaternal interface according to the species, these phenomena which, in the human species are superimposed upon classical endocrine activity, could be one of the signs of evolution. In the pregnant woman, the hypothesis of local action of signaling factors originate from intrauterine tissues in the control of uterine motility would help to at least partly understand why their placental production, like their concentration in the maternal peripheral circulation, are often impossible to correlate with the maintenance of gestation, the triggering of labor and parturition.

### BIOCHEMICAL CHANGES IN THE UTERUS AT THE END OF PREGNANCY. INTEGRATIVE PROCESSES FOR SIGNALING FACTORS

During the final weeks of pregnancy, biochemical modifications affect the uterine cervix and the myometrium. While the beginning of labor seems to occur rather suddenly "maturation" processes take place gradually, both at the level of the smooth muscle fibers (predominant in the myometrium) and that of the extracellular matrix (predominant in the cervix). The latter dissociates, which enables the cervix to dilate. Indeed, we note an increase in hydration which might be linked to that of hyaluronic acid and dispersion of collagen fibers which may be connnected to variations in the distribution of glycosaminoglycans and the release of proteolytic enzymes by cervical myofibroblasts.[9] The presence of macrophages and polynuclears may contribute, via release of lipid mediators and of cytokines, to increasing the expression of metalloproteinases, with such alterations being similar to those of an inflammatory reaction.[10] These same processes, in the myometrium, tend to favor intercellular communications (gap junctions) and the accession of smooth muscle cells toward a highly effective contractile phenotype, as witnesses by the characterization, in term myometrium, of isoforms of "marker" proteins of this phenotype (α-actin, desmin, SM1 and SM2 myosin heavy-chains) as compared to the weak presence of non-muscular isoforms.[11]

As observed for other smooth muscle cells, it is probable that such modifications affect interactions between extracellular matrix and myometrial cells via adhesion molecules (integrins, etc.).[12] These transmembrane glycoproteins, the assembly of which is regulated by a small GTP-binding protein Rho, interfere with signaling pathways and regulate cells morphology, migratory properties, growth, differentiation, and contractility.[13]

Another poorly defined aspect is that of paracrine interactions of cervical myofibroblasts and myometrial smooth muscle cells with the other cells which are found in uterus at the end of pregnancy: those of the vascular system, and in particular microcirculation, and those of the immune system (mast cells, eosinophils, neutrophils), which are important sources of signaling factors.[14] In contrast, we note the degeneration of nerve endings in the human myometrium at term.

At the moment of parturition, *control of the contractile activity of the uterine muscle* necessitates the setting up of numerous powerful regulatory systems acting not only upon expression of signals but also upon that of proteins of the signaling pathways: receptors, G proteins, effector proteins (enzymes, ionic channels, etc.) which modulate the balance between the intracellular second messengers. While inositol triphosphate (IP3) and calcium initiate contraction, cAMP and cGMP induce relaxation. These intracellular messengers can also control expression of inductible genes which help to modify the cell phenotype and consequently the responses to various stimuli.

In the human myometrium, the basic mechanisms of contraction do not appear to greatly differ from those of other smooth muscles.[15,16] The key enzyme is the myosin light-chain kinase (MLCK) which, when activated by the calcium-calmodulin complex, phosphorylates the 20-kDa myosin light-chain. In this phosphorylated form, myosin interacts with actin and induces contraction. However, when MLCK is itself phosphorylated by the protein kinases (PK) such as calcium-calmodulindependent PKII, PKC, PKA and PKG which are respectively, AMPc- and GMPc-dependent, the capacity of MLCK to activate myosin and thus to produce contraction decreases. The drop in intracellular calcium ($Ca^{2+}$i) leads to relaxation: dephosphorylated myosin, under the effect of a specific phosphatase, thus becomes detached from actin. While this schema appears to be well established *in vitro*, it does not always appear to apply as well *in vivo*. Moreover, the relative importance of the different regulatory pathways may vary according to whether they involve spontaneous contractile activity or that provoked by extracellular signals. Recent studies have underscored the fact that the streching of the muscle, which is very marked at the end of pregnancy, could stimulate the expression and the activity of enzymes of the contractile machinery, in particular by lowering the response threshold of the MLCK to $Ca^{2+}$i, which would enhance contraction. The existence of other regulatory pathways involving thin-filament binding proteins (caldesmon and calponin) is merely suggested in the myometrium.

Various specific ionic channels are expressed by the myometrium. Calcium channels, the conductance of which can be activated by a variation in transmembrane potential (VOC, or voltage-operated channels) of the L (long-lasting) type or of the T (transient) type, via attachment of a specific ligand (ROC, or receptor-operated channels) or by a mechanical strain,[17] contribute to the increase in $Ca^{2+}$i. The latter can also arise from intracellular sites whose storage capacity would increase during gestation. The sarcoplasmic reticulum, in close relationship with the caveolae of the plasma membrane, is, in the myometrium, the main site at which receptor proteins of inositol triphosphate (IP3) and of ryanodine act as channels enabling the calcium efflux necessary for contraction toward the cytoplasm. In contrast, at the level of the plasma membrane and the sarcoplasmic reticulum, several systems would contribute toward lowering the $Ca^{2+}$i, in particular the Na/Ca exchanger and the $Ca^{2+}$/ATPases which play a major role in the myometrium. These systems, which are activated by cAMP, provoke relaxation. The impact of cGMP is much less evident, as is that of the several cytosolic proteins which bind calcium.

*Activation of phospholipase C* (PLC) leads to hydrolysis of phosphatidylinositol biphosphate (PIP2) and to formation of two messengers, IP3 which mobilizes $Ca^{2+}$i and diacylglycerol (DAG), an activator of PKC. This enzymatic system is the main pathway of transduction used by the prostaglandins, oxytocin, the α1-adrenergic agents and the endothelins, to induce contraction in the human myometrium at the term.[18,19] Endothelin-1, which *in vitro* is as powerful a uterotonic agent as oxytocin,[20] is the most efficient activator of PLC.[21] This action is relayed by G proteins which are sensitive (Gi ?) and insensitive (Gq/11) to the pertussis toxin.[22] Only the endothelin receptors of the $ET_A$ type coupled with PLC are involved in contractility.[23] The proportion of these receptors increases at the end of pregnancy with respect to the $ET_B$ receptors,[26] whose function in the myometrium remains unknown.

$PGE_2$ and $PGF_2\alpha$, although they could bind to different receptors, stimulate PLC activity via a Gq/11 protein. Another G protein (Gi ?) could be implicated in the activation of the channels (ROC) by the prostaglandins.[25] The result would be a preliminary increase in $Ca^{2+}$i necessary for later activation by $PGF_2\alpha$ of a $Ca^2$-dependent PLC.[19] Conversely at the level of the uterine cervix, $PGE_2$, a powerful stimulator of cAMP synthesis by the cervical myofibroblasts, may play a crucial role in relaxation and in the processes of maturation observed at the end of pregnancy.[26] Like the endothelins, oxytocin activates the myometrial PLC via a Gq/11 protein and a G (Gi ?) protein,[27] though no preliminary influx of $Ca^{2+}$ is necessary as in the case of $PGF_2\alpha$. Nevertheless, the ROC channels could enable oxytocin to increase the $Ca^{2+}$i. The coupling of the oxytocin receptor to a Gh protein has recently been revealed in the human myometrium,[28] It is now known that it is a family of isoforms, the PLC-β, which is implicated in the activation of the receptors to seven transmembrane domains coupled to G proteins. Three isoforms, β1, β2 and β3, are involved in the contraction effect of oxytocin in the human myometrium. Another family of isoforms, PLC-γ, whose mode of activation differes from that of PLC-β, also leads to mobilization of $Ca^{2+}$i. In the human myometrium, the EGF receptor, which possesses tyrosine kinase activity, stimulates the PLC-γ via phosphorylation,[29,20] The PLC-β and PLC-γ activities would be subject to different negative retrocontrol mechanisms, generally involving PKC for PLC-β

and PKA for PLC-γ, but this aspect has not yet been elucidated in the human gravid myometrium.

In addition, oxytocin and very likely the CRH and endothelins stimulate phospholipase $A_2$ activity, leading to synthesis of endogenous prostaglandins in the human gravid myometrium. The latter, in turn, stimulate PLC-β, thereby amplifying their direct effects upon this enzymatic pathway.

In the course of the third trimester of pregnancy, *a phenomenon of heterologous desensitization* to the relaxing agents: β2-adrenergic and prostaglandins, activators of adenylyl cyclase, has been revealed in the human myometrium.[31,32]

This mechanism of desensitization also has bearing on activation of adenyly cyclase by the CRH, in the human term myometrium. Like the catecholamines and prostaglandins, the alteration in the coupling of certain of the CRH receptors to the catalytic component of adenylyl cyclase via a Gs protein has been observed.[33] In the human myometrium at term, other mechanisms contribute to lowering the cAMP concentration. Coupling of the adrenergic β2 and α2 receptors to an adenylyl cyclase inhibiting Gi protein has been observed.[31,34] The decrease in the cAMP capacities for synthesis observed at the end of pregnancy could still be accentuated at the level of its enzymatic degradation. A family of isoforms of (PDE) phosphodiesterase cyclic nucleotide which specifically degrades cAMP at high affinity, is abundant in the human myometrium at the end of pregnancy.[35] This hormone-sensitive PDE4 activity is the point of impact of pharmacologic agents with myorelaxing and anti-inflammatory properties.[36]

The nitric oxide (NO) pathway could be a link with the mechanisms responsible for maintenance of the quiescent state in the uterus during pregnancy. Indeed, its use in the treatment of threatened premature birth has been proposed. The NO synthase system, which is very active in the gravid human uterus, decreases at the time of labor. However, the role of cGMP in myometrial relaxation induced by NO has not been entirely elucidated.[37]

Other substances classically identified as growth factors (IGFs, EGF) and gonadotropins (hCG), whose receptors have recently been revealed in the human myometrium, also appear to be regulators of contraction.[38,39] This action would be due to stimulation of eicosanoïd production and/or to the fact that the multiple signals use common or interacting signaling pathways. Among various kinases, which play a role in the signaling cascade, the role of MAP kinase and tyrosine kinases needs to be clarified.[40]

Because of these phenomena of "cross-talk," the response to a given signal will vary considerably depending on the other signals present and will be an activating or an inhibiting response, probably synergic rather than additive. Moreover, it is now clear that differential control of the expression and the activity of the different protein isoforms, which intervene at each stage of the transmission of the signal, i.e., receptors, G proteins, enzymatic effectors, etc., ensure specificity and contribute to the diversity of the responses in terms of contractility.

We are now seeking to better understand which isoforms are determinant in the point of no return which, in the human myometrium at term, characterizes the onset of labor, and which factors control their expression and function. From this point of view, the steroid hormones hold a special position among the effectors of uterine activity.

It is classically considered that progesterone, due to its relaxing properties, is responsible for the state of hypocontractility of the uterus during pregnancy, in opposition to the contracting effect of 17β-estradiol. The 17β-estradiol/progesterone ratio, which is higher in the human myometrium at term than at the beginning of pregnancy, raises the question of the manner in which agonist and antagonist relationships are exerted between these two steroids, whether their effects upon uterine contraction are genomic or non-genomic.

In the myometrium as in the cervix, the steroid hormone receptors exert their pleiotropic effects by modulating the transcription of a number of target genes which code for the structural and contractile proteins, for enzymatic proteins responsible for the production of other direct or indirect effectors of uterine activity (prostaglandins, cytokines, etc.), for degradation proteins (collagenases, proteases, peptidases, phospholipases, etc.) and for various other proteins implicated in intracellular transduction (ionic channels, receptors, G proteins, etc.) and intercellular communication (connexins of the gap junctions). Results reported concerning the evolution of steroid hormone receptors in the gravid human myometrium are currently highly controversial. The most recent of these indicate that these receptors are present in the smooth muscle and the vessels of the myometrium at the end of pregnancy, and that only the progesterone receptors decrease during labor, whether or not it is premature.[41] It should be pointed out that up to now no study has been made in human myometrium of the evolution especially during pregnancy of the isoforms of progesterone and estrogen receptors, recently revealed in other tissues, which could be associated with different functions. The responses of genes modulated by the steroid hormones often necessitate the interaction of their receptors with other transcription factors. We can thus understand how difficult it is to dissociate the proper effects of the steroid hormones, whether they be direct or indirect, from those of the other hormonal signals. This difficulty is even greater due to the complexity of the relationship between the steroid hormones and their receptors, which can partly explain their complementarity and their antagonism. In the myometrium, estrogens are necessary for synthesis of the progesterone receptors, while progesterone inhibits the expression of estrogen receptors and that of its own receptors. Like other nuclear receptors referred to as "orphans," the steroid hormone receptors can be operational in the absence of a ligand.

In general, the effects of progesterone are directly opposite those of the estrogens, which induce the synthesis of signals and of proteins which intervene in contraction. Nevertheless, this is not always the case: the presence of progesterone is, in fact, required during the induction of the synthesis of certain ionic channels by 17β-estradiol. Considering that progesterone favors the expression of immunosuppressive cytokines during gestation, it remains to clarify what happens with the Th2/Th1 cytokine balance in the different cell populations of the uterus at time of parturition.

There unquestionably exist, in the myometrium, rapid non-genomic effects of steroid hormones which to a certain extent interfere via second messengers with the genomic effects. Although the notion of membrane receptors of steroid hormones remains subject to controversy, progesterone decreases and 17β-estradiol increases the fluidity of the plasma membranes.These modifications could both influence the organization and function of the membrane proteins and modulate the flexibility necessary for morphological changes which the smooth uterine fiber undergoes during the contraction-relaxation cycle. Hyperpolarizing progesterone, by inhibiting the VOC type L channels responsible for the entry of calcium into the myometrial cell and, consequently, for MLCK activity, relaxes the uterine muscle. Other calcium-calmodulin-dependent enzymes can also be inhibited, among them a family of phosphodiesterase isoforms which degrade cAMP and cGMP, both of which are involved in relaxation. In the human species, while parturition occurs with high myometrial levels of progesterone, recent experiments nonetheless suggest that the relaxing effect of progesterone upon the smooth uterine muscle is neutralized at the start of labor, or else is reversed during parturition.[42,43]

It is clearly evident at the present time that in the human species, parturition is conditioned by the progressive passage of the myometrium in a quiescent state, where the functionality of the adenylyl cyclase system predominates, toward other transduction pathways such as that involving phospholipase C, the activation of which leads to mobilization of $Ca^{2+}i$ and to contraction. Such results, when compared to those obtained in animals, underscore the specificity of the species in terms of the response of these enzymatic systems to hormonal signals in the pregnant myometrium. The impact of modifications observed in parallel at the level of the extracellular matrix upon phenotypic evolution of the uterine smooth fiber and its interactions with other uterine cells must be taken into account.

However, while all the substances present at the fetomaternal interface play a role in the uterine motility taking place during the different phases of parturition, none seems to play the initial role in setting it off. For example, prostaglandins, which exert a coordinated, efficient effect upon the myometrium and the uterine cervix, are widely used to artificially induce labor, but no element enables us to definitively state that they play a determing role in the physiological onset of parturition.

### REFERENCES

1. Challis JRG, Lye SJ: Parturition. In: Knobil E, Neill JD (eds): The Physiology of Reproduction. 2nd ed. New York: Raven Press, Ltd, pp. 985–1018, 1994.
2. Liggins GC, Fairclough RJ, Grieves SA, et al.: The mechanism of initiation of parturition in the ewe. Recent Prog Horm Res 29:111–159, 1973.
3. Ferré F, Malassiné A: Fonctions endocrines du placenta. In: Papiernik E, Cabrol D, Pons JC (eds.) Obstétrique. Flammarion: Médecine-Sciences, pp. 3–17, 1995.
4. Diczfalusy E: Steroid metabolism in the foeto-placental unit. In: Pecile, A, Finzi C (eds.): The Foeto-Placental Unit. Amsterdam: Excerpta Medica Foundation, pp. 65–109, 1969.
5. Pepe GJ, Albrecht ED: Actions of placental and fetal adrenal steroid hormones in primate pregnancy. Endocrine Rev 16:608–648, 1995.
6. Kaufman P, Burton G: Anatomy and genesis of the placenta. In: Knobil E, Neill JD (eds): The Physiology of Reproduction. 2nd ed. New York: Raven Press, Ltd, pp. 441–484, 1994.
7. Benassayag C, Mignot TM, Haourigui M, et al.: High polyunsaturated fatty acid, thromboxane A2 and alpha-fetoprotein concentrations at the human feto-maternal interface. J Lipid Res 38:71–81, 1997.
8. Pijnenborg R: Trophoblast invasion and placentation in the human: morphological aspects. Trophoblast Res 4: 33–47, 1990.
9. Cabrol D, Breton M, Berrou E, et al.: Variations in the distribution of glycosaminoglycans in the uterine cervix of pregnant women. Eur J Obstet Gynecol Reprod Biol 10:281, 1980.
10. Leppert C, Woessner JF (eds.): The Extracellular Matrix of the Uterus, Cervix and Fetal Membranes: Synthesis, Degradation and Hormonal Regulation. Ithaca, New York: Perinatology Press, 1991.
11. Cavaillé F, Fournier T, Dallot E, et al.: Myosin heavy chain isoform expression in human myometrium: presence of an embryonic nonmuscle isoform in leiomyomas and in cultured cells. Cell Motil Cytoskeleton 30:183–193, 1995.
12. Klentzeris LD: Adhesion molecules in reproduction. Br J Obstet Gynaecol 103:401–409, 1997.
13. Burridge K, Chrzanowska-Wodnicka M: Focal adhesions, contractility, and signaling. Ann Rev Cell Dev Biol 12:463–518, 1996.
14. Rudolph MI, de los Angeles Garcia M., Sepulveda M, et al.: Ethodin: pharmacological evidence of the interaction between smooth muscle and mast cells in the myometrium. J Pharmacol Exp Ther 292:256–261, 1997.
15. Carsten ME, Miller JD (eds.): Uterine Function. Molecular and cellular aspects. New York: Plenum Press, 1990.
16. Barany M (ed): Biochemistry of smooth muscle contraction. San Diego: Academy Press, xxvi, 1996.
17. Smith PG, Tokui T, Ikebe M: Mechanical strain increases contractile enzyme activity in cultured airway smooth muscle cells. Am J Physiol 268:L999–L1005, 1995.
18. Breuiller-Fouché M, Héluy V, Fournier T, et al.: Endothelin receptors: binding and phosphoinositide breakdown in human myometrium. J Pharmacol Exp Ther 270:973–978, 1994.
19. Doualla-Bell Maka Kotto F, Breuiller-Fouché M, Geny B, et al.: Prostaglandin F2α stimulates inositol phosphate production in human pregnant myometrium. Prostaglandins 45:269–283, 1993.
20. Word RA, Kamm KE, Casey ML: Contractile effects of prostaglandins, oxytocin, and endothelin-1 in human myometrium in vitro: refractoriness of myometrial tissue of pregnant women to prostaglandins E2 and F2α. J Clin Endocrinol Metab 75:1027–103, 1992.
21. Doualla-Bell Maka Kotto F, Ferré F: Regulation of myometrial contractility in human pregnancy. In: Koppe JG, Eskes TKAB, van Geijn HP, Wiesenhaan PF, Ruys JH (eds.): Care, Concern and Cure in Perinatal Médecine, 19:131–146, 1992.
22. Héluy V, Breuiller–Fouché M, Cavaillé F, et al.: Characterization of type A endothelin receptors in cultured human myometrial cells. Am J Physiol 268:E825–E831, 1995.
23. Héluy V, Germain G, Fournier T, et al.: Endothelin ETA receptors mediate human uterine smooth muscle contraction Eur J Pharmacol 285:89–94, 1995.
24. Osada K, Tsunoda H, Miyauchi T, et al.: Pregnancy increase ET-1-induced contraction and changes receptor subtypes in uterine smooth muscle in humans. Am J Physiol 272:R541–R548, 1997.
25. Phaneuf S, Asboth G, Europe-Finner GN, et al.: Second messager pathways for oxytocin and prostaglandins in human myometrium. Biochem Soc Trans 23: 21S, 1995.
26. Carbonne B, Jannet D, Dallot E, et al.: Synthesis of glycosaminoglycans by human cervical fibroblasts in culture: effects of prostaglandin E2 and cyclic AMP. Eur J Obstet Gynecol Reprod Biol 70:101–105, 1996.
27. Ku CY, Qian A, Wen Y, et al.: Oxytocin stimulates myometrial guanosine triphosphatase and phospholipase-C activities via coupling to Gαq/II. Endocrinology 136: 1509–1515, 1995.
28. Baek KJ, Kwon NS, Lee HS, et al.: Oxytocin receptor couples to the 80 kDa $G_{h_\alpha}$ family protein in human myometrium. Biochem J 315:739–744, 1996.
29. Phaneuf S, Carrasco MP, Europe-Finner GN, et al.: Multiple G proteins and phospholipase C isoforms in human myometrial cells: implication for oxytocin action. J Clin Endocrinol Metab 81:2098–2103, 1996.
30. Carrasco MP, Phaneuf F S, Asböth G, et al.: Fluprostenol activates phospholipase C and Ca2+ mobilization in human myometrial cells. J Clin Endocrinol Metab 81:2104–2110, 1996.
31. Litime MH, Pointis G, Breuiller M, et al.: Disappearance of beta-adrenergic response of human myometrial adenylate cyclase at the end of pregnancy. J Clin Endocrinol Metab 69:1–6, 1989.
32. Litime MH and Ferré F: Evidence for reduced-prostaglandin stimulatory adenylate cyclase responses in human myometrium at the end of pregnancy. Med Sci Res 18:203–205, 1990.
33. Grammatopoulos F, Stirrat GM, Williams SA, et al.: The biological activity of the corticotropin-releasing hormone receptor-adenylate cyclase complex in human myometrium is reduced at the end of pregnancy. J Clin Endocrinol Metab 81:745–751, 1996.
34. Breuiller M, Rouot B, Litime MH, et al.: Functional coupling of the α2-adrenergic receptor adenylate cyclase complex in the pregnant human myometrium. J Clin Endocrinol Metab 70:1299–1304, 1990.
35. Leroy MJ, Cedrin I, Blanchard H, et al.: Correlation between selective inhibition of the cyclic nucleotide phosphodiesterase and the contractile activity in human pregnant myometrium near term. Biochem Pharmacol 38:9–15, 1989.
36. Leroy MJ, Lugnier C, Merezac J, Tanguy G, et al.: Isolation and characterization of the rolipram-sensitive cyclic-AMP-specific phosphodiesterase (PDE IV) in human term pregnant myometrium. Cell Signal 6:405–412, 1994.
37. Sladek SM, Magness RR, and Conrad KP: Nitric oxide and pregnancy. Am J Physiol 272:R441–R463, 1997.
38. Gargiulo AR, Khan-Dawood FS, Dawood MY: Epidermal growth factor receptors in uteroplacental tissues in term pregnancy before and after the onset of labor. J Clin Endocrinol Metab 82:113–117, 1997.
39. Zuo J, Lei ZM, Rao ChV: Human myometrial chorionic gonadotropin/luteinizing hormone receptors in preterm and term deliveries. J Clin Endocrinol Metab 79:907–911, 1994.
40. Ikebe M: Contractile mechanisms. Science 274:367–368, 1996.
41. How H, Huang Z-H, Zuo J, et al.: Myometrial estradiol and progesterone receptor changes in preterm and term pregnancies. Obstet Gynecol 86:936–940, 1995.
42. Fu X, Ulmsten U, Bäckström T: Interaction of sex steroids and oxytocin on term human myometrial contractile activity in vitro. Obstet Gynecol 84:272–277, 1994.
43. Kilarski WM, Fu X, Bäckström T, et al.: Progesterone, estradiol and oxytocin and their in vitro effects on maintening the numbert of gap junction plaques in human myometrium at term. Acta Physiol Scand 157:461–469, 1996.

## References

[PDF_00674] Baek K. J., Kwon N. S., Lee H. S., Kim M. S., Muralidhar P., Im M. J. (1996). Oxytocin receptor couples to the 80 kDa Gh alpha family protein in human myometrium.. Biochem J.

[PDF_00634] Breuiller-Fouché M., Héluy V., Fournier T., Ferré F. (1994). Endothelin receptors: binding and phosphoinositide breakdown in human myometrium.. J Pharmacol Exp Ther.

[PDF_00698] Breuiller M., Rouot B., Litime M. H., Leroy M. J., Ferré F. (1990). Functional coupling of the alpha 2-adrenergic receptor-adenylate cyclase complex in the pregnant human myometrium.. J Clin Endocrinol Metab.

[PDF_00618] Burridge K., Chrzanowska-Wodnicka M. (1996). Focal adhesions, contractility, and signaling.. Annu Rev Cell Dev Biol.

[PDF_00603] Cabrol D., Breton M., Berrou E., Visser A., Sureau C., Picard J. (1980). Variations in the distribution of glycosaminoglycans in the uterine cervix of the pregnant woman.. Eur J Obstet Gynecol Reprod Biol.

[PDF_00666] Carbonne B., Jannet D., Dallot E., Pannier E., Ferré F., Cabrol D. (1996). Synthesis of glycosaminoglycans by human cervical fibroblasts in culture: effects of prostaglandin E2 and cyclic AMP.. Eur J Obstet Gynecol Reprod Biol.

[PDF_00681] Carrasco M. P., Phaneuf S., Asbóth G., López Bernal A. (1996). Fluprostenol activates phospholipase C and Ca2+ mobilization in human myometrial cells.. J Clin Endocrinol Metab.

[PDF_00611] Cavaillé F., Fournier T., Dallot E., Dhellemes C., Ferré F. (1995). Myosin heavy chain isoform expression in human myometrium: presence of an embryonic nonmuscle isoform in leiomyomas and in cultured cells.. Cell Motil Cytoskeleton.

[PDF_00729] Fu X., Ulmsten U., Bäckström T. (1994). Interaction of sex steroids and oxytocin on term human myometrial contractile activity in vitro.. Obstet Gynecol.

[PDF_00716] Gargiulo A. R., Khan-Dawood F. S., Dawood M. Y. (1997). Epidermal growth factor receptors in uteroplacental tissues in term pregnancy before and after the onset of labor.. J Clin Endocrinol Metab.

[PDF_00693] Grammatopoulos D., Stirrat G. M., Williams S. A., Hillhouse E. W. (1996). The biological activity of the corticotropin-releasing hormone receptor-adenylate cyclase complex in human myometrium is reduced at the end of pregnancy.. J Clin Endocrinol Metab.

[PDF_00726] How H., Huang Z. H., Zuo J., Lei Z. M., Spinnato J. A., Rao C. V. (1995). Myometrial estradiol and progesterone receptor changes in preterm and term pregnancies.. Obstet Gynecol.

[PDF_00652] Héluy V., Breuiller-Fouché M., Cavaillé F., Fournier T., Ferré F. (1995). Characterization of type A endothelin receptors in cultured human myometrial cells.. Am J Physiol.

[PDF_00656] Héluy V., Germain G., Fournier T., Ferré F., Breuiller-Fouché M. (1995). Endothelin ETA receptors mediate human uterine smooth muscle contraction.. Eur J Pharmacol.

[PDF_00732] Kilarski W. M., Fu X., Bäckström T., Roomans G. M., Ulmsten U. (1996). Progesterone, oestradiol and oxytocin and their in vitro effect on maintaining the number of gap junction plaques in human myometrium at term.. Acta Physiol Scand.

[PDF_00670] Ku C. Y., Qian A., Wen Y., Anwer K., Sanborn B. M. (1995). Oxytocin stimulates myometrial guanosine triphosphatase and phospholipase-C activities via coupling to G alpha q/11.. Endocrinology.

[PDF_00704] Leroy M. J., Cedrin I., Breuiller M., Giovagrandi Y., Ferre F. (1989). Correlation between selective inhibition of the cyclic nucleotide phosphodiesterases and the contractile activity in human pregnant myometrium near term.. Biochem Pharmacol.

[PDF_00709] Leroy M. J., Lugnier C., Merezak J., Tanguy G., Olivier S., Le Bec A., Ferré F. (1994). Isolation and characterization of the rolipram-sensitive cyclic AMP-specific phosphodiesterase (type IV PDE) in human term myometrium.. Cell Signal.

[PDF_00576] Liggins G. C., Fairclough R. J., Grieves S. A., Kendall J. Z., Knox B. S. (1973). The mechanism of initiation of parturition in the ewe.. Recent Prog Horm Res.

[PDF_00685] Litime M. H., Pointis G., Breuiller M., Cabrol D., Ferre F. (1989). Disappearance of beta-adrenergic response of human myometrial adenylate cyclase at the end of pregnancy.. J Clin Endocrinol Metab.

[PDF_00638] Maka F. D., Breuiller-Fouche M., Geny B., Ferre F. (1993). Prostaglandin F2 alpha stimulates inositol phosphate production in human pregnant myometrium.. Prostaglandins.

[PDF_00659] Osada K., Tsunoda H., Miyauchi T., Sugishita Y., Kubo T., Goto K. (1997). Pregnancy increases ET-1-induced contraction and changes receptor subtypes in uterine smooth muscle in humans.. Am J Physiol.

[PDF_00589] Pepe G. J., Albrecht E. D. (1995). Actions of placental and fetal adrenal steroid hormones in primate pregnancy.. Endocr Rev.

[PDF_00663] Phaneuf S., Asboth G., Europe-Finner G. N., Watson S. P., Lopez Bernal A. (1995). Second messenger pathways for oxytocin and prostaglandins in human myometrium.. Biochem Soc Trans.

[PDF_00677] Phaneuf S., Carrasco M. P., Europe-Finner G. N., Hamilton C. H., López Bernal A. (1996). Multiple G proteins and phospholipase C isoforms in human myometrial cells: implication for oxytocin action.. J Clin Endocrinol Metab.

[PDF_00621] Rudolph M. I., de los Angeles García M., Sepulveda M., Brandan E., Reinicke K., Nicovani S., Villan L. (1997). Ethodin: pharmacological evidence of the interaction between smooth muscle and mast cells in the myometrium.. J Pharmacol Exp Ther.

[PDF_00714] Sladek S. M., Magness R. R., Conrad K. P. (1997). Nitric oxide and pregnancy.. Am J Physiol.

[PDF_00630] Smith P. G., Tokui T., Ikebe M. (1995). Mechanical strain increases contractile enzyme activity in cultured airway smooth muscle cells.. Am J Physiol.

[PDF_00642] Word R. A., Kamm K. E., Casey M. L. (1992). Contractile effects of prostaglandins, oxytocin, and endothelin-1 in human myometrium in vitro: refractoriness of myometrial tissue of pregnant women to prostaglandins E2 and F2 alpha.. J Clin Endocrinol Metab.

[PDF_00720] Zuo J., Lei Z. M., Rao C. V. (1994). Human myometrial chorionic gonadotropin/luteinizing hormone receptors in preterm and term deliveries.. J Clin Endocrinol Metab.

